# Structural and Functional Genomics of Tomato

**DOI:** 10.1155/2008/820274

**Published:** 2008-01-31

**Authors:** Amalia Barone, Maria Luisa Chiusano, Maria Raffaella Ercolano, Giovanni Giuliano, Silvana Grandillo, Luigi Frusciante

**Affiliations:** ^1^Department of Soil, Plant, Environmental and Animal Production Sciences, University of Naples “Federico II”, Via Università 100, 80055 Portici, Italy; ^2^Ente per le Nuove Tecnologie, l'Energia e l'Ambiente, Casaccia Research Center, Via Anguillarese 301, S.M. di Galeria, 00123 Roma, Italy; ^3^CNR-Institute of Plant Genetics, Via Università 133, 80055 Portici, Italy

## Abstract

Tomato (*Solanum lycopersicum* L.) is the most intensively investigated Solanaceous species both in genetic and genomics studies. It is a diploid species with a haploid set of 12 chromosomes and a small genome (950 Mb). Based on the detailed knowledge on tomato structural genomics, the sequencing of the euchromatic regions started in the year 2005 as a common effort of different countries. The manuscript focuses on markers used for tomato, on mapping efforts mainly based on exploitation of natural biodiversity, and it gives an updated report on the international sequencing activities. The principal tools developed to explore the function of tomato genes are also summarized, including mutagenesis, genetic transformation, and transcriptome analysis. The current progress in bioinformatic strategies available to manage the overwhelming amount of data generated from different tomato “omics” approaches is reported, and emphasis is given to the effort of producing a computational workbench for the analysis of the organization, as well as the functionality and evolution of the Solanaceae family.

## 1. INTRODUCTION

Tomato (*Solanum lycopersicum* L., formerly *Lycopersicon esculentum* Miller) is an economically important crop worldwide, and a preeminent model system for genetic studies in plants. It is also the most intensively investigated Solanaceous species, with simple diploid genetics, a short generation time, routine transformation technology, and availability of rich genetic and genomic resources. It has a diploid genome with 12 chromosome pairs and a genome size of 950 Mb [1] encoding approximately 35,000 genes that are largely sequestered in contiguous euchromatic regions [2]. Several resources are available for genetic/genomic research in tomato including the following: (i) tomato wild species and mutant collections; (ii) marker collections; (iii) F_2_ synteny mapping population and permanent recombinant inbred (RI) mapping populations; (iv) BAC libraries and an advanced physical map; (v) TILLING populations; and (vi) tomato microarrays, gene silenced tomato lines, and VIGS libraries (for transient silencing).

Till recently, tomato genomics has largely relied on molecular marker analysis and functional analysis of gene-sets. However, for a better understanding of structural and functional aspects of its genome, following latest high-throughput technologies are also being utilized: (i) RNA transcription and protein analysis, (ii) screening of posttranslational modifications and protein-protein interactions, and (iii) discovery of metabolic networks. The information generated by large-scale genome sequencing can lead a major revolution in the understanding of tomato biology.

The International Solanaceae Genome Project (SOL) was established to develop a network of knowledge on the Solanaceae family and to coordinate the research efforts of different groups from around the world [3]. The Solanaceae Genomics Network website (SGN;http://www.sgn.cornell.edu) was created to facilitate distribution of genomic information for tomato in particular and for Solanaceous species in general in a comparative genomic context [4]. The challenge facing SOL in the coming years is to develop methodologies that will enable genomic information to be associated with phenotypes of interest for crop improvement. The framework for organizing these data is the highly conserved genetic map of the Solanaceae that will allow the information basis to be extended beyond individual species.

Progress in tomato research will depend on our ability to tie together the independent components into higher-order complexity with multiple dimensions. Multidisciplinary research efforts, involving the increased input of chemistry, physics, statistics, mathematics, and computing sciences, are becoming increasingly crucial for the success of such approach.

## 2. STRUCTURAL GENOMICS

### 2.1. Molecular markers

Beginning in the 1980s, different types of molecular markers have been developed in tomato. Among crop species, tomato is one of the richest in the number and type of these genetic markers, including restriction fragment length polymorphisms (RFLPs), simple sequence repeats (SSRs), cleaved amplified polymorphic sequence (CAPS), amplified fragment length polymorphisms (AFLPs), and single nucleotide polymorphism (SNP). Chronologically, RFLPs were the first markers developed. Currently, more than 1000 RFLPs have been mapped on the 12 tomato chromosomes. A subset of RFLP markers has been converted into PCR-based markers through sequencing of their ends. These sequences are available from the SGN Database, thus allowing specific primers for PCR reaction to be designed. Other PCR-based markers were developed both as random markers, such as random amplified polymorphic DNA (RAPD), AFLPs, and locus-specific markers, such as SSRs, CAPS, and conserved ortholog sets (COSs); and many of them have been mapped onto the high-density tomato genetic map [5].

Given the huge number of markers that have been set up for tomato using different methods, a database collecting the different datasets is available at the SGN website. Indeed, all information for more than 15,000 markers is collected in the SGN [6], where a specific tool for “marker search” allows markers to be located on the map. Markers can be searched by name, chromosome position, mapping population, and BAC associations (if they have been associated with BAC from the tomato sequencing project by hybridization with overgo probes or computationally by BLAST comparisons). Some of them have also been grouped into collections for organizational purposes or because they are part of a particular project. So, it is possible to select either COS (markers that have been mapped in both tomato and Arabidopsis) or COSII markers (markers that have been mapped in several Asterid species, including several Solanaceous species) [7, 8]. Other groups comprise known function genes (KFG), or EST-derived (TM) markers.

Recently, large-scale sequencing work in tomato has been generating sequences of whole BAC and cloned genes, ESTs collected from different cDNA libraries, and the sequences of full-length cDNAs. The cataloguing of these sequences in public databases is providing useful information to develop markers with high resolving power, such as SNPs and InDels, thus initiating an era of in silico tomato marker discovery. The tomato SSRs are an example of genetic markers that can be mined from existing sequence data. Smulders et al. [9], by screening the EMBL and GenBank Databases, identified 36 primer pairs, which detected polymorphisms at or close to coding regions. In recent studies, as many as 2627 SSRs were mined from an EST dataset by screening the 26,363 tomato EST unigene dataset and 57,222 full-length cDNA sequences that were available in MiBASE (http://www.accelrys.com/products/gcg). Most of these SSRs (around 80%) were novel SSRs, since they did not match any of the the mapped markers, thus being candidates for novel microsatellite markers [10]. In addition, more than 250,000 ESTs derived from cDNA libraries are currently catalogued on the SGN website. All these sequences are potentially source of new markers, such as SSRs and SNPs, useful for tomato genome analysis. In fact, besides SSRs, SNPs can also be mined from sequence data [11]; and an efficient in silico SNPs discovery is feasible for tomato due to the availability of EST in public databases [12]. Moreover, in the framework of the tomato sequencing project, around 400,000 BAC ends are being sequenced that could also be mined to search SNPs among *Solanum* genotypes (Ercolano et al., unpublished results). These will allow useful PCR marker to be derived that also fall in intron regions, thus complementing the detection of polymorphism in the coding regions represented by the ESTs.

Recently, oligonucleotide-based arrays have been used to identify DNA sequence polymorphisms in different species, since they allow high-throughput development of markers. Total genomic DNA hybridization methods are also being exploited in tomato with the aim of identifying markers such as single feature polymorphisms (SFPs). For instance, a 15.27 K gene NimbleGen tomato array was used by Sim et al. [13] for a study of polymorphism between *S. lycopersicum* and its closely related wild species.

### 2.2. Genetic and physical maps

Genetic mapping of morphological traits in tomato started at the beginning of last century, and by 1973 a total of 257 morphological and disease resistance markers had been mapped [14]. By the 1990s, tomato had become one of the first plants for which RFLPs were used to generate a high-density linkage map [15]. Later several genetic maps using PCR-based markers were developed and integrated with the RFLP maps, as reviewed by Labate et al. [16]. The first PCR-based reference genetic map covering the entire tomato genome was reported by Frary et al. [5] for a population derived from the cross *S. lycopersicum* × *S. pennellii*

The Solanaceae is the first family of flowering plants for which comparative mapping was conducted [17, 18]. As a result, several genetic maps not only for tomato genome, but also for the genomes of other Solanaceous crops are now available at the SGN site [4]. Comparative genome analysis showed that tomato and potato genomes differ in only five paracentric inversions [15], whereas the tomato and pepper genomes differ in numerous rearrangements including several translocations as well as both pericentric and paracentric inversions [19, 20]. More recently, Doganlar et al. [21] have shown that eggplant and tomato genomes are differentiated by 28 rearrangements, which could be explained by 23 paracentric inversions and five translocations. These data suggest that paracentric inversions have been the primary mechanism for chromosome evolution in the Solanaceae.

Comparative genomics research is presently gaining momentum in Solanaceae due to availability of sequencing data for several species. This will greatly enhance the resolution of comparative mapping in this family. This research activity received further support due to the availability of whole genome sequence of *A. thaliana*, which facilitated the development of PCR-based COS markers using genes shared between distantly related plant taxa [7, 8]. For instance, in an effort to determine the level of synteny between *Arabidopsis* genome and the genomes of tomato and other Solanceous species, COSII markers are being mapped not only on tomato genome, but also on the genomes of other major Solanaceous species including eggplant, pepper, and *Nicotiana* (http://www.sgn.cornell.edu/markers/cosii_markers.pl). Also, in order to test the efficacy of COSII markers for comparative mapping across large phylogenetic distances, a subset of COSII markers is being mapped on the genomes of both tomato and diploid coffee (*Coffea canephora*) [8].

Besides genetic linkage maps, cytological and cytogenetic maps are also available for tomato. For example, Sherman and Stack [22] developed a physical map that was used to quantify the distribution of crossovers along each chromosome. Physical maps were also developed by in situ hybridisation techniques and allowed a comparison of linear order and distances between markers on genetic linkage maps and physical maps [23–25]. The results obtained by Peterson et al. [23] have shown that the linear order of markers is not always conserved between genetic and cytological positions.

The availability of mapped markers and of FISHed BAC allowed the construction of a high-density integrated genetic and physical map, whose definition is still in progress, and which is the foundation for the tomato genome sequencing project. Overgo analysis has been used to match BAC to probes based on markers from the *S. lycopersicon* × *S. pennellii* map. This analysis found 600 markers that unambiguously anchor over 5000 BACs to the genetic map [26]. Actually, at SGN site there are more than 10 000 BACs, which are anchored to the genetic maps.

### 2.3. QTL mapping and exploitation of natural biodiversity

The high-density RFLP linkage map developed for tomato facilitated extensive mapping of qualitative traits such as various disease resistance genes, for example, [27, 28]. This allowed tomato breeders to use marker-assisted selection (MAS) for variety improvement. Furthermore, tomato was the first species for which a whole genome molecular linkage map was used to identify quantitative trait loci (QTL) [29], leading to an understanding of the genetic basis of numerous quantitative traits including morphology, yield, fruit quality, fruit primary and secondary metabolites, as well as resistance to a variety of abiotic and biotic stresses [26]. The QTL mapping studies conducted by de Vicente and Tanksley [30] and by Eshed and Zamir [31] using mapping populations derived from interspecific tomato crosses provided stronger evidence that despite the inferior phenotype, unadapted germplasm could also be used as a source of complementary positive alleles that can result in favorable transgressive phenotypes once incorporated in the cultivated background.

The above results indicated that new molecular breeding strategies need to be devised in order to allow more efficient use of the genetic potential stored in seed banks and exotic germplasm. One such approach, the “advanced backcross QTL mapping method” was proposed by Tanksley and Nelson [32] with the purpose of combining the process of QTL analysis with variety development, by simultaneously identifying and transferring favorable QTL alleles from unadapted to cultivated germplasm. The AB-QTL strategy was initially developed and tested in tomato [33], and since then, it has been adapted for use in other crops including rice, maize, wheat, pepper, barley, and bean [34]. So far five AB-QTL studies have been conducted in tomato involving crosses with five wild *Solanum* species and in all cases favorable wild QTL alleles have been detected for more than 45% of the evaluated traits [34]. These data suggest that continued sampling of exotic germplasm should guarantee the discovery of new and useful QTL alleles. Another advantage of the AB-QTL method is that once favorable QTL alleles are detected in segregating populations (i.e., BC_2_ or BC_3_), few additional marker-assisted generations are required to develop near isogenic lines (NILs) or introgression lines (ILs) that can be phenotyped in replicated trials in order to confirm the QTL effect and subsequently be used for variety development. Numerous QTL-NILs or ILs have been developed starting from the tomato AB-QTL mapping populations, and several of them have been characterised for numerous quantitative traits, for example, [35, 36].

Since exotic germplasm is an important source of favorable alleles for the improvement of quantitative traits, introgression lines (ILs) developed in tomato have a special significance. This also supports the proposal by Zamir [37] for investment in the development of “exotic libraries.” Each such library consists of a set of ILs, each IL carrying a single marker-defined chromosome segment derived from a donor exotic parent in an otherwise homogeneous elite genetic background. The alien segment in each IL generally also carries a specific gene, preferably in homozygous condition. A set of overlapping ILs would together represent the entire genome of the donor parent and several such sets of ILs constitute a permanent genetic resource, since they can be maintained by self-pollination.

In tomato, the first exotic library ensuring whole genome coverage was developed by Eshed and Zamir [31] from the cross between the wild green-fruited species *S. pennellii* (acc. LA716) and the cultivated tomato *S. lycopersicum* (cv. M82). Presently, this library consists of 76 RFLP-defined ILs which partition the entire genetic map into 107 bins defined by single or overlapping segments [38]. Over the past 15 years, the *S. pennellii* ILs and their hybrids have been phenotyped for more than one hundred traits. For 20 different characters, such as yield, fruit morphology, and biochemical traits, repeated measurements are available, and the resulting data are presented, in silico, in a search engine called “Real Time QTL” [39] (http://zamir.sgn.cornell.edu/Qtl/Html/home.htm).

The studies conducted on the *S. pennellii* IL library have highlighted the higher QTL mapping power of “exotic libraries” compared with conventional segregating populations. Moreover, ILs have shown to be a powerful genetic tool to study the epistatic interactions among QTLs [40], to obtain more precise estimates of the magnitude of QTL x genetic background interaction [31, 36, 41], and of QTL x environment interaction [34, 36, 38, 41]. The high-resolution mapping approach applied to *S. pennellii* ILs has led to the map-based cloning of the first two QTLs ever cloned: the fruit weight QTL, *fw2.2* [42], and the sugar yield QTL, *Brix9-2-5* [43].

More recently, the *S. pennellii* “exotic library” is now being used to identify the genes that influence heterosis [44, 45]. Furthermore, MAS pyramiding of valuable wild QTLs in the genetic background of cultivated tomato has demonstrated to be a successful approach for developing breeding lines that can significantly outperform leading commercial hybrids under both wet and dry field conditions [41]. The outcome of the application of the IL breeding concept has been the development of a new processing tomato hybrid thatis currently the leading variety in California (http://www.ptab.org/ranking9.htm) (D. Zamir, personal communication).

The advantages of the IL approach have motivated the public and private sectors to invest in the development of new library resources starting from interspecific crosses with different wild species of tomato including *S. habrochaites*, *S. pimpinellifolium*, *S. lycopersicoides*, and *S. chmielewskii* [35, 46–49].

To enhance the rate of progress of introgression breeding in tomato, within the framework of a currently running EU project (EU-SOL) (http://www.eu-sol.net), a genetic infrastructure of “exotic libraries” is being further refined from a diverse selection of accessions. Moreover, the IL populations are being anchored to a common PCR marker-based framework, mostly consisting of COSII markers, which will facilitate QTL identification, mapping, cloning of the underlying genes, and the use of the novel variation in marker-assisted breeding.

## 3. STRATEGIES FOR TOMATOGENOME SEQUENCING

The tomato genome is being sequenced as the cornerstone of an International Solanaceae Genome Initiative, a project that aims at developing the family Solanaceae as a model for systems biology for understanding plant adaptation and diversification (see International Solanaceae Genome Initiative white paper (http://sgn.cornell.edu/solanaceae-project)). A sequencing strategy on a BAC by BAC basis of approximately 220 Mb euchromatin was proposed. The tomato genome comprises approximately 950 Mb of DNA—more than 75% of which is heterochromatin and largely devoid of genes [2]. Most genes are found in long contiguous stretches of gene dense euchromatin located on the distal portions of each chromosome arm. The starting points for sequencing the genome are approximately 1500 “seed” BAC clones individually anchored to the tomato map by means of overgo markers. Since most of the genetic markers anchoring the BAC correspond to genes (or ESTs), the BAC are likely to be biased towards the euchromatic portion of the genome.

**Figure 1:**
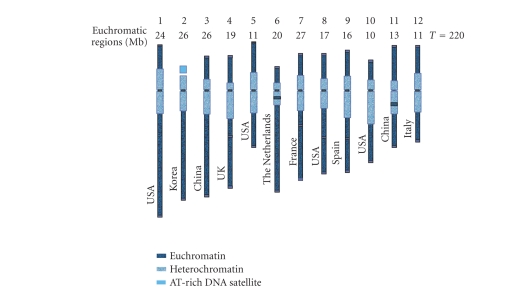
Countries partecipating to the genome sequencing project. For each chromosome the distribution of euchromatic portions is also indicated (modified from the International Solanaceae Genome Initiative white paper (http://sgn.cornell.edu/solanaceae-project).

The division of sequencing activities between countries was effected on a chromosome basis (see Figure 1). Funding agencies of each country supported the sequencing of corresponding chromosomes. Additional funds to complete this task were provided for European countries by the EU-SOL project.

In order to facilitate the sequencing task, marker analysis strategies, cytogenetic protocols, and a number of bionformatics and molecular tools have been developed in recent years. Most of the genome sequencing resources, such as BAC libraries (LE_HBa *Hin*dIII library, SL_*Mbo*I, and SL_*Eco*RI, based on the Heinz 1706 genotype) and web repositories, were provided by different partners. Seed BAC and contigs were mapped to each chromosome at Cornell University by means of overgo probes. A fingerprint map of approximately 10X genome equivalents from the LE_HBa library has been constructed at the University of Arizona through funding from the National Science Foundation (http://www.genome.arizona.edu/fpc/tomato). Recently, a Sanger Initiative was focussed on the generation of additional fingerprint data from the *Mbo*I library in order to allow comparison and integration of the two datasets. Fluorescent in situ hybridization (FISH) was provided to help guide the extension of the tiling path through the euchromatic arms of each chromosome and to determine the location of heterochromatin regions [22, 50]. Validation of single BAC assigned to each individual chromosome arm was also performed in different participant countries. Moreover, a 3D pooling library to perform new BAC screening was developed in Japan [10].

**Figure 2:**
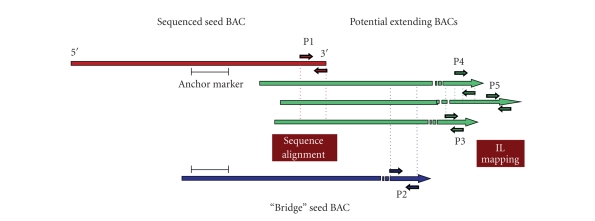
Strategy for selection of candidate extending BACs based on combined use of bioinformatics analysis and experimental molecular data. Specific primer pairs were designed on sequenced seed BAC-end (P1 pair), on “bridge” seed BAC (P2 pair), and on potential extending BACs (P3, P4, P5 pairs), selected from the BAC-end database. All primer pairs were tested to amplify fragments from candidate extending BACs. Sequences of P1 and P2 fragments were aligned to the sequenced seed BAC and to the “bridge” seed BAC, respectively, to confirm both overlapping and direction of candidate extending BACs. Amplification of P3, P4 and P5 fragments allowed the selection of the longest extending BAC. IL mapping through specific IL-12 lines confirmed the position of the selected extending BAC on chromosome 12.

Starting in 2005, during the last two years of tomato sequencing activity, the participant countries set up their own sequencing pipelines and started to construct the sequence scaffold of assigned chromosomes. Before starting the sequencing work according to mapping information available at SGN, seed BAC were selected using different strategies (IL mapping, internal sequencing strategy, FISH localization). After a low-coverage sequencing of each seed BAC, the construction of a minimal tiling path of BAC clones was performed by BLASTing the sequence of each “seed” BAC against the BAC-end STC Database to identify BAC with minimal overlap in either directions. The BAC-end Database consisting of 200,000 clones (from *EcoR*I, *Hind*III, and *Mbo*I libraries) was used both to confirm and extend the euchromatin minimal tiling path (e-MTP). Each BAC-end sequence was subjected to automated annotation to determine the proportion of ends that are likely to correspond to genic regions. To improve this process, different strategies have been developed. In the Netherlands, BAC walking was supported using a sequence-tagged connector approach based on AFLP fingerprinting as outlined in Peters et al. [51]. In Japan, selected BAC Mixture (SBM) shotgun sequencing has been set up. In this method, BAC clones whose end sequences do not contain repeat sequences are selected: then the selected BACs are mixed and shotgun sequencing is performed [10]. In our own laboratory, the use of combined bioinformatics tools and molecular data to select a minimum tiling path has been proven to reduce the overlap between adjacent clones. Good extension candidate BAC have been selected using the software “BacEnds Extension v 0.1” [52], which is complementary to the SGN Online BLAST Interface. Following selection based on bioinformatics analysis, and using the IL mapping strategy, chromosome location of the selected extending BAC was experimentally confirmed. Also, the detection of SNPs between *S. lycopersicon* and *S. pennellii* in both the resequenced anchor marker region and the BAC-ends allowed positioning of each extending BAC on chromosome 12 (see Figure 2). Despite a nonuniform distribution of seed BAC on chromosome 12, small contigs consisting of overlapping BAC started to emerge. Currently, sixty-five BACs are in different sequencing phases, and 20 of them will be available on public databases by the end of 2007. For 15 seed BAC, at least one round of extension was performed; in some cases, where two or three rounds of extension were performed, overlapping BACs were merged in sequence islands of >300 kb. The contig of approximately 500 kb between markers T1045 and T1211 was also filled up. Interestingly, the ratio of physical and genetic distance in this region is 250 kb/cM.

In all, a draft sequence has been constructed for approximately 24% of the tomato euchromatic genome space, including all the twelve chromosomes. Sequence islands spanning the genome are being joined and edited in large regions. Progress can be viewed through the development of the TPF and AGP files, available from the SGN repository. The TPF indicates the expected relative positions of the BAC and the AGP provides assembly information. The best coverage is on chromosome 2, where the sequencing of 141 BAC is finished. Large portions of chromosomes 4 (with 77 BAC already sequenced) and chromosome 8 (with 91 BAC already sequenced) have also been sequenced. In all, more than 800 BAC are in different phases of sequencing, and sequences belonging to more than 500 BAC accounting for approximately 21% of total BAC have already been submitted to the SGN website.

Assuming that work on the tomato genome project will continue at the current pace, high-quality sequencing of the euchromatic space should be completed within the next one or two years (by 2008 or 2009). Since the euchromatic portion of the genome is estimated to be approximately 220 Mb, the average physical distance between two adjacent seed BAC should be as little as 200 kb. However, the available map has insufficient density and resolution to provide a template for complete sequencing, since there are large chromosome regions, which are not yet targeted with markers. Therefore, in order to complement the ongoing sequencing project, several new strategies have been undertaken. For instance, selection of additional seed BAC with different verification methods (e.g., IL mapping, FISH, etc.) has been proposed. The recent release of markers from Syngenta to the SGN repository also allowed the identification of new candidate seed BAC, which are distributed throughout the full genome. This may prove useful for filling in gene spaces at a later stage of the project. Whole genome shotgun sequencing and the availability of new generation sequencing technologies, including 454/Roche’s sequencer FLX, Solexa’s sequencing system, and ABI’s SOLiD, may also prove useful in completing the whole genome sequencing of the tomato genome.

## 4. FUNCTIONAL GENOMICS

In order to understand the function of specific genes and their role in metabolic pathways, as also to identify the key steps in their coregulation mechanisms, several approaches have been exploited, including mutagenesis, genetic transformation, and transcriptome analysis.

### 4.1. Insertional mutagenesis

Both classical and insertional mutageneses have been used in tomato. Indeed, together with barley, *Arabidopsis*, and maize, tomato was the focus of early, extensive mutagenesis programs. In a paper published in 1964, Hans Stubbe reviewed over 250 tomato mutants arising from the seminal work of the Gatersleben group [53]. To date, over 600 characterized monogenic mutations are available in a variety of genetic backgrounds at the Tomato Genetics Resource Center (http://tgrc.ucdavis.edu). More recently, an extensive mutant population consisting of 6000 EMS-induced and 7000 fast neutron-induced mutant lines has been obtained (54). This population is probably saturating. For instance, extensive allelic tests confirmed that all the *wiry* mutants with 3 to 7 alleles present in TGRC are represented in the population. Two new *wiry* loci have also been described in the collection, each with 10 alleles. A detailed phenotypic description of the mutants is available online (http://zamir.sgn.cornell.edu/mutants).

Insertional mutagenesis systems exploiting exogenous transposon systems have also been described in tomato [55–57]. Nevertheless, these systems, some of which utilize the Micro-Tom cultivar, have not yielded saturating mutant collections and have thus not been utilized extensively. Highly efficient protocols for transformation of Micro-Tom have been described [58], which may serve as a tool for extensive T-DNA mutagenesis programs also.

#### 4.1.1. Targeting induced local lesions IN genomes

In addition to the above, a more recent strategy called targeting induced local lesions IN genomes (TILLING) was described by McCallum et al. [59] for targeting local mutations in the genome. This is, a PCR-based strategy that provides an allelic series of induced point mutations in genes of interest. As such, it can be applied to most organisms, even to those for which an efficient transformation system is not available. TILLING has been used for high-throughput isolation of mutants in Arabidopsis [60] as well as in several crop plants [61]. TILLING platforms for tomato are under development in several countries, including the US, France, Italy, and India. The Franco-Italian effort is coordinated by the EU-SOL project (http://www.eu-sol.net).

### 4.2. Gene silencing (RNAi and VIGS)

Strategies for gene silencing have also been widely used as a tool for functional genomics research in tomato. Indeed, tomato fruit ripening was one of the early systems in which both sense and antisense silencing were found to be effective [62, 63]. More recently, RNA interference (RNAi) and virus-induced gene silencing (VIGS) have also been successfully used as functional genomics tools in tomato. Interestingly, the use of RNAi remains confined in the fruit, thus making the fruit-specific silencing of genes possible [64]. Similarly, VIGS has been described in tomato roots [65] and fruits [66] although the extent to which silencing remains confined to these organs has not been extensively investigated. Several viral vectors have been used, including Tobacco rattle virus (TRV) [67], Tomato yellow leaf curl China virus isolate [68], and potato virus X [69]. Of these, TRV displays the widest host range, allowing silencing in several *Solanum* species [70], as well as in non-Solanaceous species like opium poppy [71] and *Arabidopsis* [72].

### 4.3. Transient expression of exogenous genes

Transient expression of exogenous genes has also been achieved through several transient transformation techniques, such as particle bombardment or agroinfiltration. Recently, an agroinjection technique was developed for tomato fruits [73], which allow the functional analysis of several genes in fruits in a short time. This technique has been used both for expression of exogenous genes and for TRV-induced gene silencing.

### 4.4. Transcriptional profiling

Finally, transcriptional profiling is being widely explored since the extensive EST collection available in tomato [4] has allowed designing of several microarray platforms: the most widely used to date has been Tom1, a cDNA-based microarray containing probes for approximately 8000 independent genes; and Tom2, a long oligonucleotide-based microarray containing probes for approximately 11000 independent genes. Both these microarrays are already available from BTI (http://bti.cornell.edu/CGEP/CGEP.html) and soon Tom2 will also be available from the EU-SOL project (http://www.eu-sol.net). The third array is an Affymetrix Genechip, which contains probe sets for approximately 9000 independent genes (http://www.affymetrix.com/products/arrays/specific/tomato.affx). As the tomato genome project progresses, a comprehensive, public tomato microarray platform will become indispensable.

## 5. BIOINFORMATICS

In order to address key questions arising from the SOL initiative, an overwhelming amount of data from different “*omics*” approaches is being generated and can be utilized for genomics research. Therefore, bioinformatics approaches assume major importance in order to convert raw data into biologically meaningful information. The SOL network is planning a bioinformatics infrastructure that should support integration of information from Solanaceae research into a “one-stop shop” on the web. This will ultimately allow Solanaceae biology to be approached from a systems biology perspective. The bioinformatics centers in the SOL network are all involved in building this infrastructure. It will rely on web service approaches [74] to implement a virtual online center of information dedicated to Solanaceae.

The preliminary effort of the bioinformatician in the SOL network is mainly focussed on setting up a distributed annotation pipeline to provide a high-quality, information-enriched tomato genome. For this purpose, the International Tomato Annotation Group (ITAG) has been constituted, which is a collaborative effort in annotating the tomato genome. It involves several groups from Europe, Asia, and the US. These groups of scientists are organizing data and sharing methodologies to provide a reliable tomato genome annotation. The ITAG annotation pipeline (http://www.sgn.cornell.edu/sequencing/ITAG/status_html.pl) is currently being developed through work on batches of sequences, which are generated at the SGN by grouping BAC which are being submitted by each sequencing center. Analyses, such as repeat masking, EST alignment and gene predictions, are performed on the BAC. These data are fed into the EuGene combiner software [75] for homology searches using protein or genomic sequences from other species. The resulting predicted genes are then functionally annotated based on homology searches, protein domain identifications, and Gene Ontology assignments. Each consortium member takes on different tasks according to a predefined job distribution and in accordance with its specific expertise.

The preliminary effort of a genome annotation exercise requires a definition of reliable gene models from tomato to support the training of gene predictors. The definition of a tomato-specific set of reference gene models is a necessary step towards reliable predictions and a preliminary task of the ITAG. EST/cDNA sequences can be fully exploited if they are first clustered and assembled into high-quality consensus and are then properly aligned against genomic sequences. The organization of tomato and other Solanaceae EST collections is a prerequisite to provide a preliminary annotation of the tomato genome, which is supported by experimental evidence.

Several specific EST repositories from *S. lycopersicum* are available worldwide (Table 1). The TIGR Tomato Gene Index (LeGI) is a collection of virtual tentative consensus (TC) sequences constructed by clustering and assembling 213,974 ESTs and 2,043 ETs (release 11) generated in several laboratories, including the TIGR Institute, Cornell University, and the Boyce Thompson Institute.The SOL Genomics Network (SGN) [4], a website dedicated to the biology of the Solanaceae, organizes and distributes ESTs (~239,593), sequenced from 35 different cDNA libraries from *S. lycopersicum* (32), *S. pennellii* (2), *S. habrochaites* (1), and the corresponding “combined” consensus sequences. Other EST resources are as follows: (i) the Tomato Stress EST Database (TSED), which contains ESTs from more than ten stress-treated substractive cDNA libraries from *S. lycopersicum*; (ii) the Micro-Tom Database (MiBASE) [76], which distributes unigenes obtained by assembling 35,824 Micro-Tom (a miniature and dwarf tomato cultivar) ESTs from full-length cDNA libraries and 150,581 ESTs from other tomato lines; (iii) the PlantGDB [77], which collects PlantGDB-assembled Unique Transcripts (PUT) from many different species including those of *S. lycopersicum* generated from EST sequences available at the NCBI dbEST Database [78].

CAB-developed TomatEST [79], a secondary database of EST/cDNA sequences, today contains 112 libraries from all the tomato species available at dbEST. TomatEST has been designed to provide a workbench for mining the complexity of EST sequences information content from multiple tomato species. This will then allow expression pattern analysis and gene discovery in the framework of the *S. lycopersicum* genome project.

The CAB group within the EU-SOL project (http://www.eu-sol.net) is committed to collect all EST data from Solanaceae species available in dbEST (Table 1) and to provide EST alignments to the tomato genome draft sequences under production. Also ESTs from two species of the *Coffea* genus (Rubiaceae) were considered in the CAB collection, since coffee and tomato share common gene repertoires [80]. A specific tool has been designed to remove the overrepresented EST sequences from each of the 16 species collections in order to clip the original datasets and produce nonredundant sets of sequences. These EST collections are independently processed by the ParPEST pipeline [81] to generate *tentative consensus sequences* (TCs).

The definition of gene models based on EST/cDNA data is a complex effort. D’Agostino et al. [82] proposed a methodology based on the analysis of spliced alignments of EST and tentative consensus sequences to automatically define a reliable dataset of gene models in tomato. Suitable methodologies are used for EST clustering and assembling, and for EST/TC to genome mapping; and resulting conflicts and ambiguities are independently classified.

In the current update of the tomato genome, 582 TCs of *S. lycopersicum* have been selected as they are consistently supported by EST evidence [81]. Among these TCs, 70 cover at least 95% of the length of the most similar protein sequence; 168 cover at least 50%; 257 cover less than 50% of the matching protein, and the remaining 87 show no significant similarity to known proteins. Considering TCs from other tomato species among those mapping to the *S. lycopersicum* BAC, additional 21 loci are located. The number accordingly increases to 251 loci if the potato TC sequences are also considered. Only the TCs covering 95% of the length of the matching protein are selected as *complete* reliable gene models for training gene predictors. To date they account for a total of 108 gene models.

In order to contribute to the tomato genome annotation and to accomplish the requirements of an efficient data integration, the CAB group developed ISOL@ (Chiusano et al., unpublished results), an Italian bioinformatics resource for Solanaceae genomics. This effort is conceived to support the analysis of the genome organization and its functionality in the light of evolutionary approaches over the entire Solanaceae family.

ISOL@ is currently organized into two main levels: the *genome* level and the *expression* level. The cornerstone of the genome level is represented by the tomato genome draft sequences. The founding elements of the expression level are the Solanaceae EST collections and the oligonucleotide probe sets, which have been generated for the production of the tomato expression microarrays (http://www.affymetrix.com/products/arrays/specific/tomato.affx). A nonstop crosstalk between the levels is based on data source sharing and on integration of the information, which belongs to the respective under parts. Each level can be independently accessed through specific web interfaces which allow user-driven data investigation (http://biosrv.cab.unina.it/isola/isola.html).

In order to provide a preliminary annotation of the BAC sequences while waiting for the whole genome annotation that will be provided by the ITAG, the CAB group has set up an automated annotation pipeline in order to ensure daily retrieval of new S. lycopersicum BAC sequences from GenBank, which are used to feed the genome annotation process. The BAC annotation process aims at identifying genes and other genetic elements on the draft genome sequence. The protein coding “gene finding” process is exclusively based on spliced-alignments of expressed sequence tags (ESTs) to the *S. lycopersicum* genomic sequences that are also provided to the distributed pipeline, which was set up within the ITAG effort. To accomplish this task, ESTs from the different plant source collection at CAB (Solanaceae and Rubiaceae species), and the corresponding TCs, created by assembling ESTs in a cluster, are both used. Noncoding RNAs (ncRNAs) from the Rfam collection [83] are also aligned to the genomic sequences. The TIGR Solanaceae Repeats Database [84] is the resource selected for the identification of repetitive sequences in the *S. lycopersicum* genome. The Italian platform also includes alignments of all the RNA sequences from Arabidopsis to the tomato genomic sequences in order to identify genes that are conserved between the two species.

The collection, as of July 2007, comprises 186 BAC sequences which have been uploaded from all the sequencing centers to the GenBank Database. The BAC sequences collected and annotated are released to the scientific community through the Gbrowse [85] web application at CAB (http://biosrv.cab.unina.it/GBrowse). Tracks showing annotations and other features are displayed and cross-linked with other local or external databases which can be explored through web interfaces.

This above effort aims at producing a computational workbench for the analysis of the organization, the functionality, and the evolution of genomes in the Solanaceae family. In addition, it provides experimental biologists with a preliminary annotation of tomato genome data, and represents a reference point while sequencing the tomato genome. Indeed, the crosstalk between the sequencing data and the computationally defined TCs may support BAC extension [86] and a preliminary evaluation of the gene content of each BAC under sequencing.

## 6. PERSPECTIVES

In the “-omics” age, strategies for integrated genomics that include DNA sequence mining, expression profiling data, and functional and molecular diversity analyses of candidate genes, combined with the use of introgression lines, can increase the efficiency in discovery, candidate gene identification, and cloning of target QTLs [87]. Given the large amount of data that is being generated and will be generated in future, a current bioinformatics challenge is to develop user-friendly bioinformatics management systems that will allow description of the genetic components of subtle QTLs and their integration with genome information including gene content, expression, and function [41]. The development of tomato into a model Solanaceous plant, with a large collection of genetic and genomic tools and a high-quality reference genome sequence, and the high throughput sequencing of 100 additional Solanaceae genomes (SOL-100 project) will facilitate the understanding of the incredible ecological and morphological adaptation exhibited by the Solanaceae family.

## Figures and Tables

**Table 1:** Solanaceae EST resources in the world

Resource	Web address	Species included

Solanaceae Genomics Network (SGN)	www.sgn.cornell.edu	Tomato, potato, pepper, eggplant, and petunia

TIGR Plant Gene Indices	compbio.dfci.harvard.edu/tgi/plant.html	Tomato, potato, pepper, petunia, tobacco, and *N. benthamiana*

PlantGDB—Plant Gene Indices	www.plantgdb.org/prj/ESTCluster/index.php	Tomato, potato, petunia, and different species of *Nicotiana* genus

Tomato Stress EST Database (TSED)	ibs.sinica.edu.tw/ibsdb/app_all/index.php	Tomato ESTs from stress-treated substractive cDNA libraries

MIBASE—Micro-Tom Database	www.kazusa.or.jp/jsol/nicrotom/indexj.html	Micro-Tom EST libraries

Italian Solanaceae EST Database	biosrv.cab.unina.it	Tomato, potato, and other Solanaceae species EST collections
